# Demand for and use of modern contraception among young women aged 15–24 years in Malawi: evidence from the Malawi demographic health survey, 2015–2016

**DOI:** 10.3389/frph.2025.1719985

**Published:** 2025-11-24

**Authors:** Redson Mwandama, Sydney Nkhoma, Steven Henry Dunga

**Affiliations:** 1 Department of Economics, University of Malawi, Zomba, Malawi; 2 Department of Agriculture and Applied Economics, Lilongwe University of Agriculture and Natural Resources, Lilongwe, Malawi; 3 School of Economic Sciences, North-West University, Vanderbijlpark, South Africa

**Keywords:** contraception use, modern contraception, demand satisfied (mDFPS), unmet need, parity

## Abstract

**Introduction:**

This study examined the determinants of the demand for and use of modern contraception among young women aged 15–24 years in Malawi, focusing on the met demand for family planning with modern methods (mDFPS). Previous studies in Malawi have primarily assessed contraceptive prevalence or intentions to use, while limited attention has been given to mDFPS, a key indicator of reproductive autonomy.

**Data and methods:**

The analysis used data from the 2015–16 Malawi Demographic and Health Survey (MDHS). After excluding cases with missing values, the final analytical sample comprised 7,643 young women aged 15–24 who had a demand for family planning. Weighted descriptive, bivariate, and probit regression analyses were conducted to identify factors associated with modern contraceptive use, demand for contraception, and mDFPS.

**Results:**

Bivariate analysis showed significant associations between mDFPS and age, education, marital status, parity, employment, and exposure to health workers (*p* < 0.001). Multivariate probit regression indicated that higher education, employment, and later sexual debut increased the probability of mDFPS by 11%, 3%, and 7%, respectively, while being married reduced it by 14% (*p* < 0.01). Parity remained the strongest predictor, with women having one or more children being 44%–55% more likely to have mDFPS. Factors such as wealth, residence, and information exposure were not significant after adjustment.

**Conclusion:**

By focusing on mDFPS rather than overall contraceptive prevalence, this study advances understanding of reproductive autonomy among young Malawian women. The findings suggest that educational attainment, empowerment, and gender-sensitive service delivery are more decisive than economic access alone. Efforts to improve mDFPS should therefore address social norms, marital dynamics, and health system barriers to youth-friendly contraceptive services.

## Introduction

Access to modern contraception remains a significant global public health and development challenge. Despite notable advancements in expanding family planning services, approximately 218 million women in low- and middle-income countries (LMICs) who wish to avoid pregnancy are not using modern contraceptives (1, 2). Each year, around 121 million pregnancies are unintended, with more than half occurring in LMICs, where access barriers persist (3, 4). These barriers are systemic and multifaceted, including frequent shortages of essential contraceptives at primary health care clinics due to supply chain weaknesses, which limit the availability of preferred methods (5, 6). Logistical challenges, such as long distances to health facilities and high transportation costs, further impede consistent access to services for women, particularly in rural areas (7, 8). Policy and operational hurdles, including restrictive regulations and the inadequate integration of family planning with other health services, further exacerbate these access issues (9, 10). For adolescents and young women aged 15–24, the consequences of unmet needs are severe, leading to early and unintended pregnancies, unsafe abortions, and school dropouts. Pregnancy-related complications remain one of the leading causes of death among adolescent girls globally (11). These outcomes not only compromise women's health and rights but also reduce productivity and hinder economic growth.

In sub-Saharan Africa, fertility decline has been uneven, and the unmet need for contraception remains one of the highest in the world, with more than 20% of women experiencing unmet need in at least 15 countries (12). While contraceptive prevalence exceeds 70 per cent in high-income regions, it remains low across much of sub-Saharan Africa (13) with a pooled estimate of 18.36% (95% CI: 18.48) among married women, ranging from 5.04% in Chad to 59.79% in Lesotho (14), and 29.6% among women with an average fertility rate (12). The regional total fertility rate averages above 4.5 children per woman, nearly double the global average (15). Progress has been constrained by persistent social and cultural barriers, misinformation, and limited access to youth-friendly services (16). Many adolescents continue to experience stigma when seeking contraceptive services, and health providers often discourage use among unmarried young women (17). The result is a wide gap between contraceptive awareness and actual use.

While in Malawi, the national modern contraceptive prevalence reached 58% among married women in 2015–16 (18). The 2024 report from the National Statistics Office shows continued gains with 66% of current married women using modern methods (19). However, this national success masks disparities among subgroups, particularly young women aged 15–24. Uptake remains as low as 24% among adolescents and 42% among unmarried sexually active youth (20, 21). Qualitative and mixed-methods studies highlight persistent barriers, including provider bias, lack of privacy, myths about infertility, and partner disapproval, particularly in rural areas and during crises (22–24). While community-based distribution and youth-friendly initiatives have expanded access, stock-outs, limited method choice, and judgmental attitudes undermine effectiveness (25, 26). Recent evidence from Phalombe and Blantyre underscores that although fertility awareness is high, social norms and health system gaps continue to drive inconsistent and covert use among adolescent girls (22, 27).

Prior research in Malawi and sub-Saharan Africa has examined contraceptive prevalence and intentions to use (20, 21), yet few studies quantify the gap between expressed demand and its satisfaction with modern methods—a gap that directly measures reproductive autonomy. While these are important metrics, they do not fully capture the concept of reproductive autonomy, which is the ability to effectively act upon one's fertility preferences. A more nuanced indicator, the met demand for family planning with modern methods (mDFPS), has emerged as a critical benchmark. This indicator measures the proportion of women with a demand for family planning whose demand is actually satisfied by modern contraception. It directly quantifies the success of a health system in translating women's needs into effective service utilization.

This study advances the existing discourse by shifting the analytical focus from contraceptive prevalence to the critical gap between demand and utilization, using the met demand for family planning (mDFPS) indicator. This provides a more holistic assessment of reproductive autonomy. Furthermore, we integrate economic and behavioural perspectives within a socio-ecological framework. While data constraints require the use of proxy variables for some constructs, this approach allows us to investigate how individual characteristics, interpersonal dynamics, and broader structural factors intersect to influence contraceptive outcomes. These contributions bridge theoretical and empirical gaps and inform targeted interventions to reduce unmet need.

### Theoretical and conceptual framework

Contraceptive behaviour can be understood as a product of both economic reasoning and behavioural influences. In the model of fertility economics, individuals and households make reproductive decisions by balancing the costs and benefits of childbearing. As education and employment opportunities rise, the opportunity cost of having children increases, leading to higher demand for contraception (28). This framework explains how economic empowerment can alter fertility preferences and encourage contraceptive use.

However, economic factors alone do not fully capture reproductive behaviour. Behavioural and psychosocial theories add depth to this understanding. The Theory of Planned Behaviour (29) suggests that contraceptive use depends on intentions shaped by attitudes, subjective norms, and perceived control. Similarly, the Health Belief Model (30) posits that individuals adopt health behaviours when they perceive benefits to outweigh risks and barriers. The Ecological Systems Theory (31) further situates these decisions within a wider environment, where family, peers, community, and institutions interact to influence outcomes.

Integrating these perspectives, this study adopts a socio-economic and behavioural framework in which a young woman's ability to meet her contraceptive demand with modern methods depends on both individual capacities and external constraints. The framework considers six interrelated domains: Demographic and socioeconomic factors such as age, education, residence, and wealth influence exposure to information and opportunity costs of fertility; Fertility experience and preferences shape motivation to delay or limit childbearing; Information and media exposure affect knowledge and attitudes toward contraception; Empowerment and decision-making power determine a woman's ability to act on her preferences; Health system contact and service quality influence accessibility, privacy, and provider attitudes; Sexual behaviour and exposure determine the immediacy of contraceptive need.

**Figure 1:**
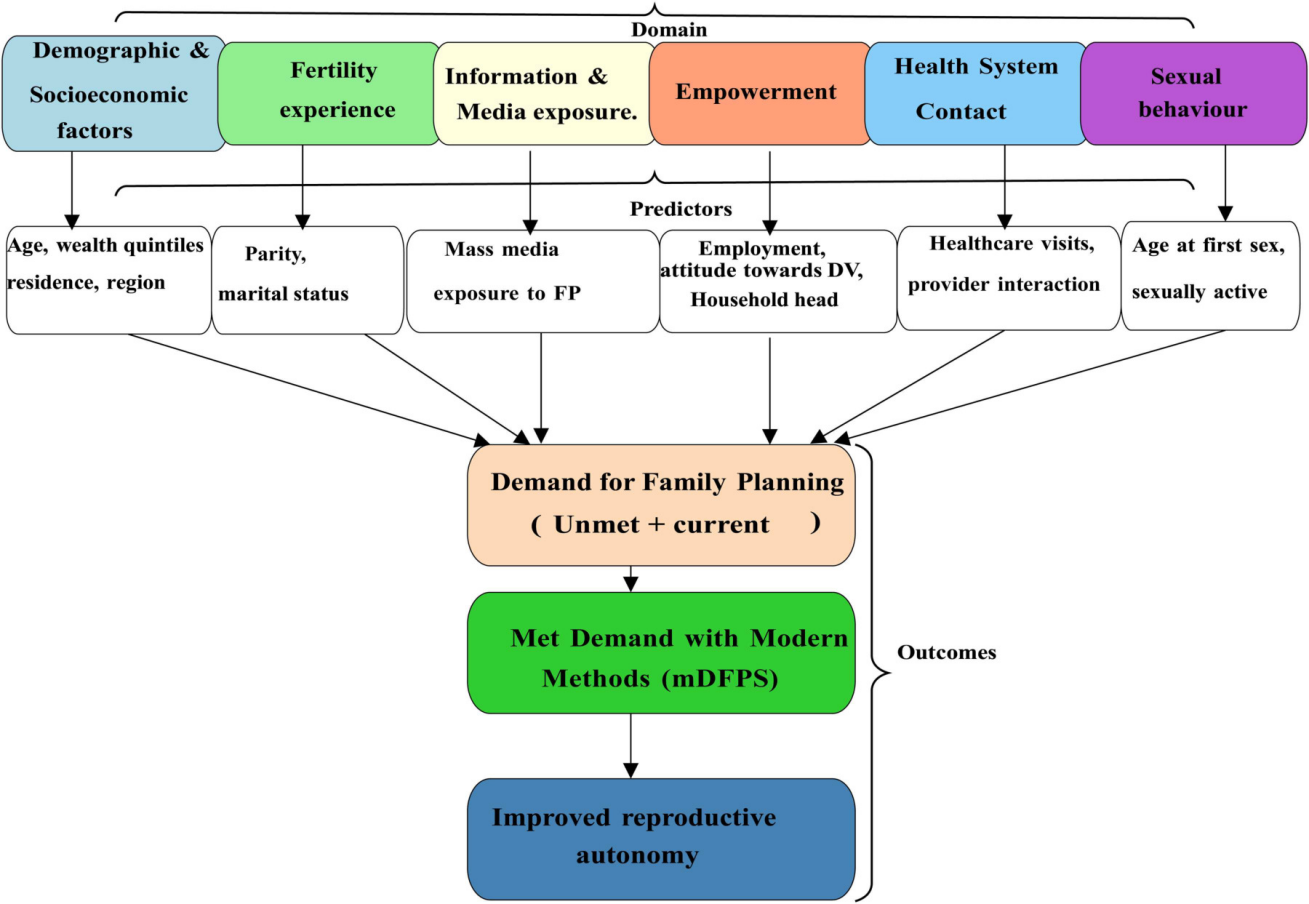
Conceptual framework of the predictors of modern contraceptive demand and Use among young women in Malawi.

Together, these factors determine the likelihood that a woman's demand for family planning is satisfied with modern methods. Figure 1 illustrates the Conceptual Framework of the Predictors of Modern Contraceptive Demand and Use among Young Women in Malawi. This framework illustrates how individual, interpersonal, and structural factors interact to influence the likelihood that young women's demand for family planning is satisfied with modern methods (mDFPS). The framework integrates economic and behavioural theories, showing that socioeconomic conditions, fertility preferences, information, empowerment, and health system factors operate through interrelated pathways to shape contraceptive behaviour.

### Literature review

Globally, advancing and advocating for sexual reproductive health among young girls and women has attracted enormous attention from policymakers and scholars. One major determinant of good access to sexual reproductive health is the unmet need for women (32). reiterate that contraception, coupled with the unmet need for women in family planning strategies are significant driver for measuring access to sexual reproductive health. In particular, comprehensive algorithms have been developed to effectively measure and quantify the unmet need for proper policy interventions (33).

In order to have a complete understanding of the unmet need concept among women, other scholars have assessed the spatial heterogeneities and the factors that affect the unmet need for spacing among women (34). The study indicates a higher spatial variation across India, and advocate for increasing women's education towards unmet need for spacing, similarly echoed by (35, 36).

Empirical evidence from sub-Saharan Africa consistently highlights the complex interplay of social, economic, and cultural factors that influence contraceptive demand and use among young women. These determinants can be organized into five main themes: socioeconomic factors, education and employment, gender and cultural norms, access to information and services, and empowerment.

Socioeconomic and demographic factors are strong predictors of contraceptive use. Multicountry studies across Africa show that education, household wealth, and urban residence are positively associated with modern contraceptive uptake (37, 38). Women in rural areas often face logistical barriers such as long distances to clinics and poor service quality, while urban women have better access and exposure to family planning messages (39). However, some evidence suggests that wealth differences are narrowing where contraceptives are provided free through public or NGO programs, as in Malawi (25).

Education and employment play central roles in shaping reproductive choices. Higher education levels are associated with improved knowledge of contraceptive methods, greater autonomy, and delayed marriage (40, 41). Employment increases women's financial independence and bargaining power, allowing them to negotiate contraceptive use more effectively (42). From a health economics perspective, both education and employment raise the opportunity cost of unplanned childbearing, thereby encouraging investment in contraception.

Gender and cultural norms remain powerful constraints. In many societies, early marriage, pressure to prove fertility, and patriarchal decision-making limit young women's autonomy (43, 44). Social stigma surrounding premarital sex discourages unmarried adolescents from seeking contraception (17, 45). In Malawi, cultural expectations around motherhood and moral judgment from providers exacerbate these barriers (27). As a result, even when young women have demand for contraception, social approval and partner acceptance often determine actual use.

Information and health system access are also crucial. Although knowledge of contraception is high, misinformation about side effects and infertility persists (22). Health system challenges, including stock-outs, limited method choice, and lack of privacy, further reduce uptake (23). Studies have shown that outreach by health workers can increase awareness but is less effective when services are perceived as judgmental or not youth-friendly (46, 47). Quality of counselling and confidentiality are thus as important as physical access.

Empowerment and autonomy are consistently linked to higher contraceptive use. Women who participate in household decisions or reject gender-based violence are more likely to use modern methods (48). Empowerment enhances self-efficacy, confidence, and negotiation capacity within relationships. In Malawi, however, many young wives lack control over reproductive decisions due to economic dependency or fear of conflict with partners (49). Efforts to strengthen empowerment through education, gender-transformative programs, and community dialogue have been shown to improve uptake and continuation of contraception.

The evidence across sub-Saharan Africa shows that modern contraceptive use increases with education, employment, and empowerment, but declines under restrictive gender norms and weak service delivery. However, few studies have examined the met demand for family planning with modern methods (mDFPS) a key indicator of reproductive autonomy among young women in Malawi. Most existing research focuses on contraceptive prevalence or intention rather than the satisfaction of demand. This study addresses this gap by analyzing the determinants of both demand and met demand using nationally representative data, integrating economic and behavioral perspectives to explain how individual and structural factors jointly shape contraceptive outcomes among Malawian youth.

## Methodology

### Data source and sample size

This study utilised data from the 2015–2016 Malawi Demographic and Health Survey (MDHS), a nationally representative cross-sectional survey conducted by the National Statistical Office of Malawi in collaboration with ICF International (18). The MDHS employed a stratified, two-stage cluster sampling design, with enumeration areas as primary sampling units and households as secondary units, to provide reliable estimates for key demographic and health indicators.

The total MDHS sample comprised 24,562 women aged 15–49 years who were interviewed. For the present analysis, the sample was first restricted to young women aged 15–24 years, resulting in an initial subsample of 10,367 individuals. Following standard DHS procedures for constructing family planning indicators (Cardona et al., 2025; S.E.K. and J.B., 2014), women were classified as having a demand for family planning if they were currently using any contraceptive method (modern, traditional, or folkloric) or had an unmet need for spacing or limiting births. Among the 10,367 young women, 9,103 (87.8%) reported having a demand for family planning.

To ensure data quality and completeness for the multivariate models, cases with missing or inconsistent values on key explanatory variables—particularly age at first sex, number of health personnel visits in the past 12 months, and other core covariates—were excluded using listwise deletion, as applied in previous DHS-based studies (50, 51). This step reduced the sample to 8,373 young women with non-missing data. Finally, restricting to those with demand for family planning yielded the final analytical sample of 7,643 young women (91.3% of the 8,373 with complete data).

**Figure 2:**
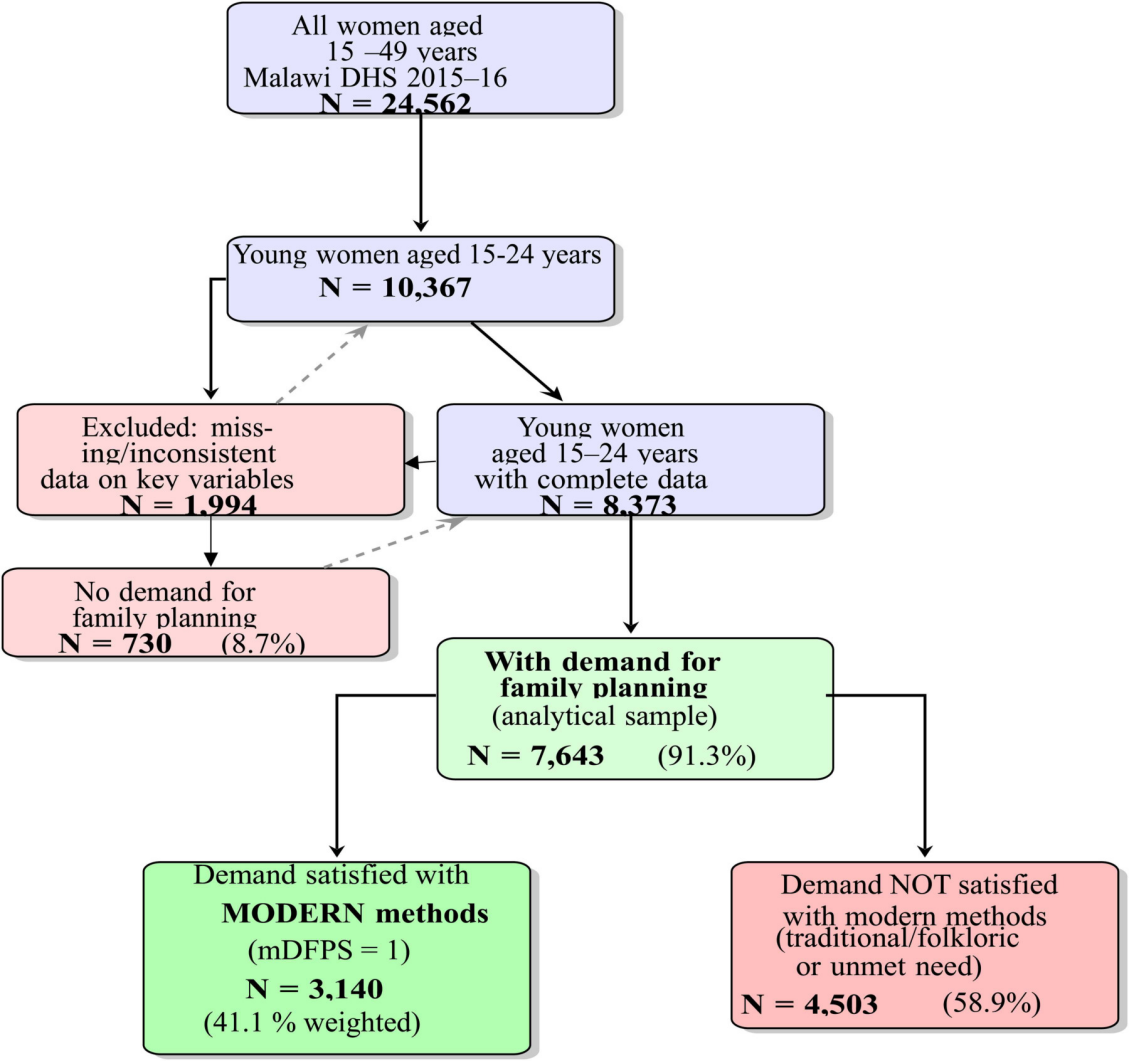
Flowchart on derivation of the analytical sample on demand for family planning with modern methods (mDFPS) among women aged 15–24 years, Malawi demographic and health survey, 2015–16.

All descriptive statistics, bivariate associations, and probit regression models presented in this study are based exclusively on these 7,643 young women who had a demand for family planning and complete data on all variables of interest. Survey weights, clustering, and stratification were applied throughout to account for the complex survey design and ensure nationally representative estimates, See Figure 2.

### Construction of the dependent Variable

The construction of the dependent variable followed DHS guidelines step by step to ensure replicability. First, current contraceptive users were identified using the DHS contraceptive method variable. Women reporting use of modern methods, including pills, injectables, implants, intrauterine devices, sterilisation, or condoms, were coded as modern users. Those using traditional methods (such as withdrawal or periodic abstinence) or folkloric methods were flagged separately.

Next, the unmet need for family planning was derived from the DHS fertility preferences module, which indicates whether a woman desires to delay or limit childbearing but is not currently using contraception. A binary variable “demand for family planning” was then created: a woman was coded as having demand if she was a current user of any method (modern, traditional, or folkloric) or reported unmet need. Those with neither method use nor unmet need were coded as not having demand.

Finally, the mDFPS variable was defined within this analytic subpopulation of women with demand. A respondent was coded 1 if she had demand and was using a modern method, and 0 if she had demand but was either using traditional/folkloric methods or had an unmet need. Women without demand were excluded from the denominator (52–55). This construction ensures that the indicator captures the proportion of women whose demand for family planning is actually satisfied with modern contraception.

Formally, let *Di* denote demand (1 = demand, 0 = no demand) and *Mi* denote use of a modern method (1 = modern, 0 = otherwise). Then:

$$\mathrm{mDFP}S_{i}=\{\begin{array}{cc} 1 & \mathrm{if}\;D_{i}=1\;\mathrm{and}\;M_{i}=1\\ 0 & \mathrm{if}\;D_{i}=1\;\mathrm{and}\;M_{i}=0\\ \mathrm{missing} & \mathrm{if}\;D_{i}=0 \end{array}.$$

Thus, the dependent variable is binary, taking the value 1 when demand is satisfied by modern contraception and 0 when demand is unsatisfied or met only by traditional methods. This explicit definition makes clear that women using traditional or folkloric methods are considered to have a demand but are unsatisfied with modern means**.**

### Explanatory variables

Explanatory variables were constructed from standard DHS recode variables. These variables have been emphasized by different authors (20, 34, 36) to influence the demand and use of contraceptives in Malawi and beyond. These variables include Age (15–19 and 20–24 years), place of residence (urban, rural), educational attainment (no education, primary, secondary and tertiary), household wealth was proxied by the DHS wealth quintile (poor, middle and rich), Marital status (single and married), parity (0, 1, 2–4 and 5 + children), access to information (yes, no), employment status (employed, not employed), attitudes toward domestic violence (accepts some form of domestic violence, rejects all domestic violence), health care visits (yes, no), age at first sex (*<* 16, 16–19, 20 + years), sexually active (yes, no), geographical region (North, Central and South) and household head (male, female).

Although the conceptual framework outlines multi-level influences on contraceptive behaviour, not all factors are directly observable in the DHS dataset. To reflect these broader dimensions, proxy indicators were used. Structural and health system factors were captured through variables such as region of residence, type of household headship, and contact with health facilities. Community-level effects were represented by region and rural–urban residence, which approximate cultural and infrastructural differences across local contexts. Interpersonal factors were incorporated through marital status, partner-related variables, and decision-making autonomy within households. This operationalisation ensures that the empirical analysis remains aligned with the multi-level theoretical model despite data limitations.

### **Model** specification

Given the binary nature of the outcome, a probit regression model was employed. Let the latent propensity to use modern contraception be

$$Y_{i}^{*}=X_{i}\beta +\;\varepsilon_{i},\varepsilon_{i}∼\;N\;(0,\;1),$$

With observed outcome:

$$Y_{i}=\{\begin{array}{cc} 1 & \mathrm{if}\;Y_{i}^{*}>0\\ 0 & \mathrm{otherwise} \end{array}$$

The conditional probability of adopting modern contraception is therefore

$$P(Y_{i}=1|\;X_{i})=\;\Phi (X_{i}\beta )$$

where *Φ* is the cumulative distribution function of the standard normal distribution.

Because probit coefficients are not directly interpretable, marginal effects were computed to assess substantive importance. For continuous covariates, the marginal effect is

$$\frac{\partial P(Y_{i}=1|\;X_{i})}{\partial X_{ik}}=\phi (X_{i}\beta )\beta_{k}$$

where ϕ is the standard normal density function. For binary covariates, marginal effects were estimated as discrete probability changes:

$$\Delta =\Phi (X_{i}^{D=1}\beta )-\Phi (X_{i}^{D=0}\beta )$$

averaged across the sample. We emphasize average marginal effects (AMEs), calculated as

$$\mathrm{AM}E_{k}=\frac{1}{N}\;\sum_{i=1}^{N}\phi (X_{i}\hat{\beta }){\hat{\beta }}_{k}$$

This approach allows interpretation of results in terms of probability differences, which are more meaningful for policy.

### Modelling strategy

To reflect the sequential nature of the theory of change, the model was specified in blocks. The baseline included demographic and socioeconomic characteristics. Fertility experience and preferences were then added. Information exposure was introduced in the third model. Women's empowerment was included in the fourth model. The full specification added health system contact and sexual exposure. This nested modelling strategy mirrors the hypothesized pathways in the theory of change, allowing assessment of the incremental explanatory power of each block of variables.

Sampling weights, clustering, and stratification from the DHS design were incorporated to ensure nationally representative inference. Standard errors for marginal effects were derived using the delta method.

## Results

**Figure 3:**
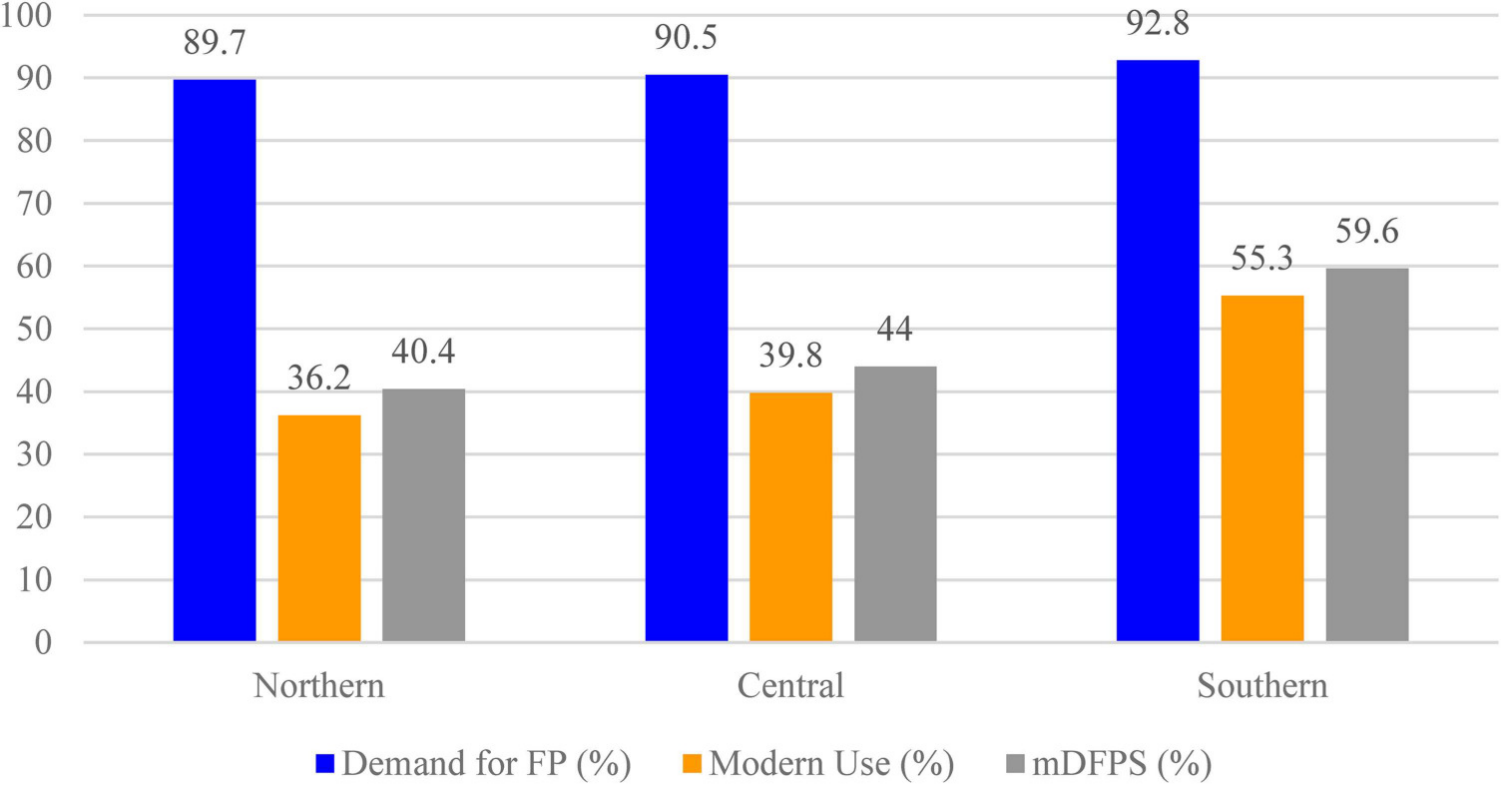
Regional distribution of demand for family planning (%), modern contraceptive use (%), and met demand for family planning with modern methods (mDFPS, %) among young women aged 15–24 years in Malawi, 2015–2016 MDHS.

Figure 3 illustrates the regional distribution of demand for family planning (FP), modern contraceptive use, and met demand for family planning with modern methods (mDFPS) among young women aged 15–24 years in Malawi based on the 2015–2016 Malawi Demographic and Health Survey (MDHS). The demand for FP is highest in the Southern region at 92.8%, followed by the Central region at 90.5%, and the Northern region at 89.7%. In terms of modern contraceptive use, the Northern region has the lowest rate at 36.2%, with the Central region at 39.8%, and the Southern region at the highest rate of 55.3%. As a result, mDFPS shows a clear gradient from south to north: 59.6% in the Southern region, 44.0% in the Central region, and 40.4% in the Northern region.

### Background characteristics

Table 1 outlines the socio-demographic, economic, and reproductive characteristics of 7,643 young women with demand for family planning. The majority of participants were aged 20–24 years (62.5%). A significant proportion lived in rural areas (80.0%) and had completed primary education (64.5%), with secondary education attained by 28.6%. In terms of wealth distribution, 41.0% belonged to the poor quintile, while 40.7% were in the rich quintile. Geographically, most respondents were from the Southern region (48.2%). Early sexual activity was notably higher among those aged 16 to 19 years (52.5%). And Only 13.3% had been visited by a health field worker in the past year, while 81.4% rejected all forms of domestic violence. Slightly more than half of the participants were employed (52.8%) and sexually active (52.0%), and 64.4% had access to family planning information. Parity was low, with 32.6% having no children, 38.7% having one child, 28.6% having 2–4 children, and just 0.2% having five or more. Notably, most of the women were married (70.8%), and 72.0% lived in male-headed households.

**Table 1:** Socio-demographic, economic, and reproductive characteristics of young women aged 15–24 years with demand for family planning (analytical sample, *N* = 7,643), Malawi DHS 2015–16.

Characteristics (*n* = 7,643)	*N*	Percent
mDFPS		
Demand not met by modern	4,503	58.9
Demand met by modern	3,140	41.1
Age group		
15–19	2,870	37.6
20–24	4,773	62.5
Residential area		
Rural	6,116	80.0
Urban	1,527	20.0
Education level		
No Education	373	4.9
Primary	4,929	64.5
Secondary	2,185	28.6
Tertiary	156	2.0
Wealth quintile		
Poor	3,137	41.0
Middle	1,399	18.3
Rich	3,107	40.7
Region		
Northern	1,360	17.8
Central	2,599	34.0
Southern	3,684	48.2
Age at first sex		
Less than 16 years	3,131	41.0
16–19 years	4,012	52.5
20+ years	500	6.5
Visited by a health field worker		
Yes	1,015	13.3
No	6,628	86.7
Attitude on domestic violence		
Accepts some DV	1,424	18.6
Rejects all DV	6,219	81.4
Employment status		
Not employed	3,608	47.2
Employed	4,035	52.8
Sexually active		
Yes	3,975	52.0
No	3,668	48.0
Access to information		
Yes	4,920	64.4
No	2,723	35.6
Parity		
0 children	2,494	32.6
1 child	2,955	38.7
2–4 children	2,182	28.6
5+ children	12	0.2
Marital status		
Single	2,232	29.2
Married	5,411	70.8
Household head		
Male	5,500	72.0
Female	2,143	28.0

### Bivariate associations of mDFPS with background characteristics

Table 2 presents the percentage of young women whose demand for family planning was satisfied with modern methods (mDFPS) based on selected characteristics, along with the results of chi-square tests. Bivariate analysis indicated significant associations between mDFPS and various factors. Women aged 20–24 were nearly twice as likely to have mDFPS (49.0%) compared to adolescents (27.9%; *p* < 0.001). mDFPS was higher among women with primary education (43.1%) than among those with no education (36.7%), secondary education (38.2%), or tertiary education (29.5%; *p* < 0.001). Unexpectedly, mDFPS decreased with increasing wealth, from 44.6% in the poorest quintile to 37.3% in the richest (*p* < 0.001). Regional differences were marginally significant (*p* = 0.060), with the highest mDFPS observed in the Southern (59.9%) and Central (57.1%) regions, compared to the Northern region (40.3%). Women who experienced a later sexual debut (ages 16–19: 43.0%) had higher mDFPS than those with an earlier debut (39.1%; *p* = 0.002). Exposure to health field workers was associated with increased mDFPS (49.8% vs. 39.7%; *p* < 0.001), as was employment (46.9% vs. 34.6%; *p* < 0.001) and sexual activity (55.6% vs. 25.4%; *p* < 0.001). Access to information showed a modest positive association (42.2% vs. 39.1%; *p* = 0.008). mDFPS rose sharply with parity, increasing from 11.8% among nulliparous women to 60.3% among those with 2–4 children (*p* = 0.001). Married women had a higher mDFPS (49.7%) compared to single women (20.1%; *p* = 0.001), and women in male-headed households had higher mDFPS (44.9%) than those in female-headed households (31.3%; *p* = 0.001). No significant differences were found based on rural-urban residence (*p* = 0.194) or attitudes toward domestic violence (*p* = 0.473).

**Table 2:** Percentage of young women whose demand for family planning is satisfied with modern methods (mDFPS) by selected characteristics, Malawi DHS 2015–16 (*N* = 7,643, women with demand for family planning).

Characteristics (*n* = 7,643)	N	mDFPS		Chi2 significance test
Age group		Yes	No	*P* < 0.001
15–19	2,870	27.9	72.1	
20–24	4,773	49.0	51.0	
Residential area				
Rural	6,116	41.5	58.6	*P* = 0.194
Urban	1,527	39.6	60.4	
Education level				*P* < 0.001
No Education	373	36.7	63.3	
Primary	4,929	43.1	56.9	
Secondary	2,185	38.2	61.8	
Tertiary	156	29.5	70.5	
Wealth quintile				*P* < 0.001
Poor	3,137	44.6	55.4	
Middle	1,399	41.7	58.3	
Rich	3,107	37.3	62.7	
Region				*P* = 0.060
Northern	1,360	40.3	59.7	
Central	2,599	57.1	42.9	
Southern	3,684	59.9	40.1	
Age at first sex				
Less than 16 years	3,131	39.1	60.9	*P* = 0.002
16–19 years	4,012	43.0	57.0	
20+ years	500	38.4	61.6	
Visited by health field worker				
Yes	1,015	49.8	50.2	*P* < 0.001
No	6,628	39.7	60.3	
Attitude on domestic violence				
Accepts some DV	1,424	40.2	59.8	*P* = 0.473
Rejects all DV	6,219	41.3	58.7	
Employment status				
Not employed	3,608	34.6	65.4	*P* < 0.001
Employed	4,035	46.9	53.1	
Sexually active				
Yes	3,975	55.6	44.4	*P* < 0.001
No	3,668	25.4	74.7	
Access to information				
Yes	4,920	42.2	57.8	*P* = 0.008
No	2,723	39.1	60.9	
Parity				
0	2,494	11.8	88.2	*P* = 0.001
1	2,955	51.6	48.4	
2–4	2,182	60.3	39.7	
5+	12	58.3	41.7	
Marital status				
Not married	2,232	20.1	79.9	*P* = 0.001
Married	5,411	49.7	50.3	
Household head				
Male	5,500	44.91	55.09	*P* = 0.001
Female	2,143	31.26	68.74	

### Probit regression results

The results in Table 3 show that the likelihood of using modern contraceptives is positively influenced by several factors: education (ME = 0.14, *p* < 0.000), having 1 child (ME = 0.44, *p* < 0.000) or 2–4 children (ME = 0.55, *p* < 0.000), employment opportunities (ME = 0.02, *p* < 0.000), age at first sexual encounter (ages 16–19: ME = 0.06, *p* < 0.000; ages 20 and older: ME = 0.10, *p* < 0.000), being sexually active (ME = 0.24, *p* < 0.000), and geographical location (central region: ME = 0.02, *p* < 0.000; southern region: ME = 0.03, *p* < 0.000). Conversely, the demand for contraceptives is negatively associated with individuals aged 20–24 (ME = −0.06, *p* < 0.000). However, demand is positively influenced by being in a female-headed household (ME = 0.03, *p* < 0.000) and residing in the Southern Region (ME = 0.08, *p* < 0.000). Regarding mDFPS, the study found negative associations for individuals aged 20–24 (ME = −0.04, *p* < 0.05), those living in urban areas (ME = −0.14, *p* < 0.000), and in female-headed households (ME = −0.03, *p* < 0.1).

**Table 3:** Marginal effects from probit models of modern contraceptive use, demand for contraception, and met demand for family planning with modern methods (mDFPS) among young women aged 15–24 years, Malawi DHS 2015–16 (*N* = 7,643).

Variables	Modern contraceptive user (1)	Demand for contraceptives (2)	mDFPS (3)
Age-group 15–19 (reference)			
Age—Group 20–24	−0.03** (0.01)	−0.06** (0.03)	−0.04** (0.01)
Education level			
No Education (reference)			
Primary education	0.08*** (0.02)	−0.03 (0.07)	0.11*** (0.02)
Secondary education	0.11*** (0.02)	−0.12 (0.07)	0.15*** (0.03)
Tertiary education	0.14*** (0.03)	−0.17* (0.09)	0.18*** (0.05)
Wealth quintile			
Poor (reference)			
Middle	0.01 (0.01)	0.03 (0.02)	0.01 (0.02)
Rich	−0.01 (0.01)	−0.03 (0.02)	−0.01 (0.02)
Marital Status			
Not Married (reference)			
Married	−0.08*** (0.01)	—	−0.14*** (0.02)
Residence area			
Rural (reference)			
Urban	0.010 (0.01)	−0.00 (0.02)	0.01 (0.02)
Parity			
0 (reference)			
1	0.44*** (0.02)	—	0.44*** (0.02)
2–4	0.55*** (0.02)	—	0.55*** (0.02)
5+	0.36 (0.28)	—	0.32 (0.28)
Household head			
Male (reference)			
Female	−0.06 (0.01)	0.03** (0.02)	−0.03* (0.01)
Access to information			
No (reference)			
Yes	0.01 (0.01)	0.04 (0.02)	0.02 (0.01)
Employed status			
Not employed (reference)			
Employed	0.02*** (0.01)	−0.02 (0.02)	0.03*** (0.01)
Attitude on domestic violence			
Accept some DV (reference)			
Reject all DV	0.00 (0.01)	0.02 (0.02)	0.00 (0.01)
Visit by a health facility worker			
No (reference)			
Yes	0.004 (0.01)	0.06 (0.04)	0.00 (0.02)
Age at first sex			
Less than 16 years (reference)			
16–19 years	0.06*** (0.01)	—	0.06*** (0.01)
20+ years	0.1*** (0.02)	—	0.07*** (0.03)
Sexually active			
No (reference)			
Yes	0.24*** (0.01)	—	0.26*** (0.01)
Region			
Northern (reference)			
Central region	0.02* (0.01)	0.03 (0.02)	0.03* (0.02)
Southern region	0.03** (0.01)	0.07*** (0.02)	10.03** (0.02)

Standard errors in parentheses.

*** *p* < 0.01.

** *p* < 0.05.

* *p* < 0.1.

## Discussion

This study reveals that the demand for and use of contraceptives among girls and young women are influenced by various factors. These factors, deeply rooted in African and Asian geographical contexts, provide a crucial policy foundation for enhancing the uptake of contraceptives and effectively regulating population growth.

The findings indicate that women aged 20–24 are 2.5 percentage points less likely to use modern contraception and 6.0 percentage points less likely to express a demand for contraception compared to those aged 15–19. This is somewhat unexpected, as older youth generally exhibit higher levels of sexual activity and autonomy. However, in Malawi, many women in their early twenties are already married or cohabiting and face social pressure to demonstrate fertility early in their marriages. Research by (20, 21) shows that young wives often encounter partner opposition and have limited decision-making power, which discourages contraceptive use. Consequently, while adolescents may be more interested in delaying their first births, older youth face relational and cultural barriers that hinder both their demand for and use of contraception.

Education emerges as a significant driver of modern contraceptive use and (mDFPS). Women with primary education are 7.7 percentage points more likely to use modern contraception and 10.9 percentage points more likely to meet their demand compared to uneducated women. This effect increases to approximately 17.8 percentage points among those with tertiary education. Education enhances reproductive knowledge, communication skills, and self-efficacy, empowering women to make informed and independent choices about fertility. It also delays marriage and fosters career or academic aspirations, reducing the incentive for early childbearing (27, 37). These results align with findings by (40, 56), which demonstrate that education is a powerful enabler of reproductive autonomy. In the context of Malawi, improving girls' education remains a fundamental step toward reducing unmet need and achieving universal access to family planning.

Wealth does not significantly correlate with any of the three outcomes, indicating that economic status alone does not influence contraceptive behavior among young women. Malawi's extensive network of public and NGO health services provides contraceptives at no cost, mitigating financial disparities (25). However, as noted by (57), social stigma, misinformation, and fear of side effects often play a more significant role than financial barriers. Therefore, the findings emphasize that social and informational obstacles are the primary deterrents to contraceptive use among Malawian youth.

Marriage significantly decreases the likelihood of using modern contraception (–8.4 points) and mDFPS (–13.6 points). This pattern reflects the paradox of marriage, which serves as both a legitimate context for sexual activity and a barrier to contraceptive autonomy. In Malawi's patriarchal culture, husbands often dominate reproductive decision-making, and contraception may be perceived as mistrustful or inappropriate within marriage (43, 49). Many young wives also avoid health facilities due to fear of community judgment. Consequently, marriage acts as a constraint rather than a facilitator of contraceptive uptake among young women.

There are no significant differences in contraceptive outcomes between urban and rural women, likely indicating improved physical access through community-based distribution programs (25). Nevertheless, qualitative evidence from (47) suggests that rural youth still encounter privacy concerns and limited method choices. Thus, the results may obscure deeper issues related to service quality and comfort in seeking care rather than mere physical distance from facilities.

Parity is one of the strongest determinants across all models. Having one child increases the likelihood of using modern contraception by 43.5 percentage points, and having two to four children increases it by 54.9 points, with similar increases observed for mDFPS. This suggests that, in Malawi, contraceptive use is primarily motivated by desires for spacing rather than the prevention of first births. After having one or two children, women become more aware of the demands of childcare and are more likely to adopt family planning (21, 38). The effect diminishes for women with five or more children, likely due to fertility completion or sample size limitations.

The study suggests that living in a female-headed household increases contraceptive demand by approximately 3.2 points, although it slightly decreases modern contraceptive use and (mDFPS). While female headship may foster openness about reproductive decisions, it does not necessarily ensure adequate access to resources or social support to fulfill that demand. Research by (48) indicates that many female-headed households result from widowhood or migration, which can diminish economic security and access to services. This finding highlights the complexity of empowerment and its varied effects on reproductive behavior.

Access to family planning information shows a positive but statistically insignificant relationship with all three outcomes. This implies that merely receiving information about contraception is insufficient; the quality, accuracy, and tone of that information are crucial. In Malawi, young individuals often encounter abstinence-focused or moralistic messages that lack practical contraceptive knowledge (24, 45). Without credible, youth-centered communication, exposure to information alone does not lead to behavioral change.

Employment is positively and significantly associated with both modern contraceptive use (+2.3 points) and mDFPS (+3.2 points). Employed young women tend to have greater financial independence and confidence in decision-making, which facilitates contraceptive uptake. This aligns with findings by (42, 48), who noted that employment enhances agency and negotiation power in fertility choices. In Malawi, informal employment also broadens women's social networks, exposing them to peers who normalize the use of modern contraceptive methods.

Regarding attitudes toward domestic violence, rejecting all forms of domestic violence, such as wife-beating, has a positive but statistically insignificant relationship with contraceptive outcomes. This suggests that while attitudinal empowerment matters, it does not automatically translate into practical autonomy in male-dominated households (44). observed similar trends, noting that entrenched patriarchal norms can undermine individual beliefs, limiting behavioral change even among empowered women. Visits from health workers show no significant effect across any models. Although outreach programs in Malawi are widespread, young women frequently report uncomfortable interactions with providers, including moral judgment or breaches of confidentiality (23). This indicates that the issue lies not in coverage but in the quality of interpersonal care (46). Youth-friendly services that prioritize privacy, empathy, and nonjudgmental counselling could enhance the effectiveness of these visits.

Furthermore, the study finds that delayed sexual debut significantly increases both modern contraceptive use and mDFPS. Women who initiate sexual activity between the ages of 16 and 19 are 6.3 points more likely to use contraception, while those who begin at 20 or older are 9.9 points more likely to do so. Later initiation typically indicates higher education levels and increased reproductive self-efficacy, whereas early initiation is often unplanned and associated with coercion or lack of knowledge (22). These results underscore the necessity of comprehensive sexuality education to empower youth with the knowledge and confidence to make informed choices before becoming sexually active.

Sexual activity emerges as one of the strongest predictors across all three models. Being sexually active increases the likelihood of modern contraceptive use by 23.7 points and mDFPS by 25.8 points. This finding intuitively suggests that sexually active women are more motivated to prevent unintended pregnancies. It also reflects pragmatic behavior among unmarried youth who discreetly use contraception to avoid social stigma and disruptions to their education or employment (58, 59).

Regional effects, while small, are significant. Women in the Central and Southern regions are 2–3 points more likely to use modern contraception or meet their demand compared to those in the Northern region. This trend may reflect stronger family planning programs and NGO partnerships in the South, while the North maintains more conservative cultural and religious attitudes toward contraception, as noted by (47, 57). These regional differences emphasize the need for localized interventions that are sensitive to cultural diversity.

### Policy recommendations

The findings from this study highlight the need for policy interventions addressing various socio-economic factors. First, the study advocates for increased access to education for girls and young women to enhance their sexual and reproductive health. Making education free could boost enrollment, leading to greater awareness of family planning methods. Additionally, the study calls for improved access to contraceptive information across different regions in Malawi. This could be achieved by establishing youth clubs focused on sexual and reproductive health. In areas where youth face barriers, radio programs can effectively increase the demand for contraceptives. Moreover, targeted initiatives are necessary to challenge harmful gender norms, especially within marriage, through community dialogues and campaigns that involve men and boys as partners in reproductive health. The health system needs a fundamental shift toward youth-responsive services, which includes training providers in non-judgmental and confidential counselling, as well as ensuring a consistent supply of a diverse range of contraceptive methods to meet varying preferences and needs. Furthermore, comprehensive sexuality education programs should be strengthened to reach adolescents before they become sexually active, equipping them with the knowledge and skills for informed decision-making. Lastly, interventions must be customized to address local cultural contexts and the specific barriers identified in regions with lower performance, ensuring that national successes in contraceptive prevalence benefit all subgroups of young women equitably.

## Conclusion

This study highlights a significant and ongoing gap between the demand for modern contraception and its actual use among young women aged 15–24 in Malawi, despite widespread awareness of contraceptive methods. The analysis indicates that contraceptive behavior is influenced not just by access, but by a complex interplay of socio-ecological factors. Key predictors such as higher education, employment, and later initiation of sexual activity greatly enhance both the demand for and the effective use of modern contraception, emphasizing the empowering influence of education and economic independence. In contrast, marriage poses a considerable obstacle, reflecting the strong impact of patriarchal norms and partner dynamics that limit autonomous decision-making for young wives. The positive relationship between the number of children and contraceptive use suggests that motivation is primarily driven by the need for birth spacing after the first child, rather than by the desire to prevent pregnancy before the first birth. Interestingly, factors like wealth, urban living, and general information exposure have a limited effect, indicating that structural barriers such as stigma, provider attitudes, and the quality of interpersonal care are more critical than just physical or financial access. Although regional variations exist, they are modest, underscoring the necessity for context-specific interventions. Ultimately, these findings demonstrate that enhancing contraceptive uptake requires a comprehensive approach that goes beyond supply-side solutions to address the deep-seated social, relational, and health system barriers that disproportionately impact young women's reproductive autonomy.

## Data Availability

Publicly available datasets were analyzed in this study. This data can be found here: The data supporting the findings of this study are publicly available from the Demographic and Health Surveys (DHS) program at https://dhsprogram.com. Researchers can apply for access through the DHS data request portal. But for this study, you are free to contact the corresponding author to share the data.

## References

[1] Coulson J Sharma V Wen H. Understanding the global dynamics of continuing unmet need for family planning and unintended pregnancy. China Popul Dev Stud. (2023) 7(1):1–14. 10.1007/s42379-023-00130-7 37193368 PMC10075166

[2] Pillai VK Nagoshi JL. Unmet family planning need globally: a clarion call for sharpening current research frame works. Open Access J Contracept. (2023) 14:139–47. 10.2147/OAJC.S378042 37492186 PMC10364818

[3] Baker D Sedgh G Keogh S Luchsinger G Roseman M Solo J SEEING tHE UNSEN_the Case for Action in Theneglected Crisis Ofunintended Pregnancy. New York: UNFPA (2022).

[4] Bearak J Popinchalk A Ganatra B Moller AB Tunçalp Ö Beavin C Unintended pregnancy and abortion by income, region, and the legal status of abortion: estimates from a comprehensive model for 1990–2019. Lancet Glob Heal. (2020) 8(9):e1152–61. 10.1016/S2214-109X(20)30315-6 32710833

[5] Ashipala DO Lifalaza A. Exploring the factors contributing to contraceptive stock-outs at primary health care clinics in North-Eastern Namibia. African J Reprod Heal Rev Africaine la Santé Reprod. (2025) 29(7):36–47. 10.29063/ajrh2025/v29i7.4 40728196

[6] Olaniran A Briggs J Pradhan A Bogue E Schreiber B Dini HS Stock-outs of essential medicines among community health workers (CHWs) in low-and middle-income countries (LMICs): a systematic literature review of the extent, reasons, and consequences. Hum Resour Health. (2022) 20(1):58. 10.1186/s12960-022-00755-8 35840965 PMC9287964

[7] Varela C Young S Mkandawire N Groen RS Banza L Viste A. Transportation barriers to access health care for surgical conditions in Malawi a cross sectional nationwide household survey. BMC Public Health. (2019) 19(1):264. 10.1186/s12889-019-6577-8 30836995 PMC6402149

[8] Dawkins B Renwick C Ensor T Shinkins B Jayne D Meads D. What factors affect patients’ ability to access healthcare? An overview of systematic reviews. Trop Med Int Heal. (2021) 26(10):1177–88. 10.1111/tmi.13651 34219346

[9] Cohen MA Gold S Ostrega A Zingbagba M. National policy influences of contraceptive prevalence and method mix strategy: a longitudinal analysis of 59 low-and middle-income countries, 2010–2021. Glob Heal Sci Pract. (2024) 12(2):e2300352. 10.9745/GHSP-D-23-00352 PMC11057802 38604782

[10] Shinde S Yelverton C Ali NB Partap U Ouédraogo M Yusufu I Integrating family planning with nutrition and other sexual and reproductive health services in low-income and middle-income countries: findings from a scoping review. BMJ Glob Heal. (2025) 10(Suppl 1):e017482. 10.1136/bmjgh-2024-017482 PMC12128479 40447308

[11] WHO. Adolescent pregnancy fact sheet (2014). p. 1. Available online at: https://www.who.int/news-room/fact-sheets/detail/adolescent-pregnancy (Accessed October 10, 2025).

[12] Ahinkorah BO Budu E Aboagye RG Agbaglo E Arthur-Holmes F Adu C Factors associated with modern contraceptive use among women with no fertility intention in sub-saharan Africa: evidence from cross-sectional surveys of 29 countries. Contracept Reprod Med. (2021) 6(1):22. 10.1186/s40834-021-00165-6 34332644 PMC8325798

[13] Blackstone SR Nwaozuru U Iwelunmor J. Factors influencing contraceptive use in sub-Saharan Africa: a systematic review. Int Q Community Health Educ. (2017) 37(2):79–91. 10.1177/0272684X16685254 28056643

[14] Tesema ZT Tesema GA Boke MM Akalu TY. Determinants of modern contraceptive utilization among married women in sub-Saharan Africa: multilevel analysis using recent demographic and health survey. BMC Womens Health. (2022) 22(1):181. 10.1186/s12905-022-01769-z 35585626 PMC9118760

[15] Adde KS Dickson KS Ameyaw EK Amo-Adjei J. Contraception needs and pregnancy termination in sub-Saharan Africa: a multilevel analysis of demographic and health survey data. Reprod Health. (2021) 18(1):177. 10.1186/s12978-021-01227-3 34454510 PMC8403371

[16] Bain L E Amu H Enowbeyang Tarkang E. Barriers and motivators of contraceptive use among young people in sub-Saharan Africa: a systematic review of qualitative studies. PLoS One. (2021) 16(6):e0252745. 10.1371/journal.pone.0252745 34086806 PMC8177623

[17] Kibira SPS Karp C Wood SN Desta S Galadanci H Makumbi FE Covert use of contraception in three sub-Saharan African countries: a qualitative exploration of motivations and challenges. BMC Public Health. (2020) 20(1):865. 10.1186/s12889-020-08977-y 32503485 PMC7275340

[18] Adolph R. The national statistical office. Malawi Demogr Heal Surv 2024. (2016) 3(2024):1–23. Available online at: http//:doi.org.www.nsomalawi.mw (Accessed October 10, 2025).

[19] NSO. Malawi Demographic and Health Survey 2024: Key Indicators Report. Zomba, Malawi/Rockville, Maryland, USA (2024). Available online at: https://cms.nsomalawi.mw/api/download/487/2024-MDHS-KIR-Final.pdf (Accessed October 10, 2025).

[20] Forty J Rakgoasi SD Keetile M. Patterns and determinants of modern contraceptive use and intention to usecontraceptives among Malawian women of reproductive ages (15–49 years). Contracept Reprod Med. (2021) 6(1):21. 10.1186/s40834-021-00163-8 34193289 PMC8247247

[21] Mandiwa C Namondwe B Makwinja A Zamawe C. Factors associated with contraceptive use among young women in Malawi: analysis of the 2015–16 Malawi demographic and health survey data. Contracept Reprod Med. (2018) 3(1):12. 10.1186/s40834-018-0065-x 30250748 PMC6146597

[22] Hajison PL Mpachika-Mfipa F Pitso L Tshotetsi L Chimatiro CS. Fertility awareness, perceived factors and approaches to improve contraceptive uptake among sexually active adolescent girls in Phalombe, Malawi: a mixed-methods study. Reprod Health. (2024) 21(1):161. 10.1186/s12978-024-01904-z 39533398 PMC11555923

[23] Burke HM Mkandawire P Phiri MM Kachale F Little K Bakasa C Documenting the provision of emergency contraceptive pills through youth-serving delivery channels: exploratory mixed methods research on Malawi’s emergency contraception strategy. Glob Heal Sci Pract. (2024) 12(5):e2400076. 10.9745/GHSP-D-24-00076 PMC11521556 39362664

[24] Bhushan NL Fisher EB Maman S Speizer IS Gottfredson NC Phanga T Communication, social norms, and contraceptive use among adolescent girls and young women in Lilongwe, Malawi. Women Health. (2021) 61(5):440–51. 10.1080/03630242.2021.1917479 33941050 PMC8182971

[25] Masiano SP Green TL Dahman B Kimmel AD. The effects of community-based distribution of family planning services on contraceptive use: the case of a national scale-up in Malawi. Soc Sci Med. (2019) 238:112490. 10.1016/j.socscimed.2019.112490 31437769

[26] Barden-O’Fallon J Evans S Thakwalakwa C Alfonso W Jackson A. Evaluation of mainstreaming youth-friendly health in private clinics in Malawi. BMC Health Serv Res. (2020) 20(1):79. 10.1186/s12913-020-4937-9 32013943 PMC6998314

[27] Dombola GM Manda WC Chipeta E. Factors influencing contraceptive decision making and use among young adolescents in urban Lilongwe, Malawi: a qualitative study. Reprod Health. (2021) 18(1):209. 10.1186/s12978-021-01259-9 34663362 PMC8524908

[28] Becker GS. An economic analysis of fertility. In: Universities-National Bureau Committee for Economic Research, editors. Demographic and Economic Change in Developed Countries. New York: Columbia University Press (1960). p. 209–40.

[29] Ajzen I. The theory of planned behavior. Organ Behav Hum Decis Process. (1991) 50(2):179–211. 10.1016/0749-5978(91)90020-T

[30] Rosenstock IM. Historical origins of the health belief model. Health Educ Monogr. (1974) 2(4):328–35. 10.1177/109019817400200403 299611

[31] Bronfenbrenner U. The Ecology of Human Development: Experiments by Nature and Design. Cambridge: Harvard University Press (1979). p. 352.

[32] United Nations. World Contraceptive Use. World Contraceptive Use | Population Division (2024).

[33] Bradley SEK Casterline JB. Understanding unmet need: history, theory, and measurement. Stud Fam Plann. (2014) 45(2):123–50. 10.1111/j.1728-4465.2014.00381.x 24931072 PMC4369378

[34] Rahaman M Rana MJ Roy A Chouhan P. Spatial heterogeneity and socio-economic correlates of unmet need for spacing contraception in India: evidences from national family health survey, 2015–16. Clin Epidemiol Glob Heal. (2022) 15(September 2021):101012. 10.1016/j.cegh.2022.101012

[35] Nyarko SH. Spatial variations and socioeconomic determinants of modern contraceptive use in Ghana: a Bayesian multilevel analysis. PLoS One. (2020) 15(3):e0230139. 10.1371/journal.pone.0230139 32155217 PMC7064218

[36] Rahaman M Singh R Chouhan P Roy A Ajmer S Rana MJ. Levels, patterns and determinants of using reversible contraceptives for limiting family planning in India: evidence from national family health survey, 2015–16. BMC Womens Health. (2022) 22(1):1–13. 10.1186/s12905-022-01706-0 35439954 PMC9020013

[37] Apanga PA Kumbeni MT Ayamga EA Ulanja MB Akparibo R. Prevalence and factors associated with modern contraceptive use among women of reproductive age in 20 African countries: a large population-based study. BMJ Open. (2020) 10(9):e041103. 10.1136/bmjopen-2020-041103 32978208 PMC7520862

[38] Abdulai M Kenu E Ameme D Bandoh D Tabong P Lartey A Demographic and socio-cultural factors influencing contraceptive uptake among women of reproductive age in Tamale Metropolis, Northern Region, Ghana. Ghana Med J. (2020) 54(2):64–72. 10.4314/gmj.v54i2s.11 33536671 PMC7837347

[39] Oumer M Manaye A Mengistu Z. Modern contraceptive method utilization and associated factors among women of reproductive age in Gondar City, Northwest Ethiopia. Open Access J Contracept. (2020) 11:53–67. 10.2147/OAJC.S252970 32612400 PMC7322113

[40] Alemayehu GA Fekadu A Yitayal M Kebede Y Abebe SM Ayele TA Prevalence and determinants of contraceptive utilization among married women at dabat health and demographic surveillance system site, northwest Ethiopia. BMC Womens Health. (2018) 18(1):118. 10.1186/s12905-018-0611-3 29970089 PMC6029026

[41] Bhushan NL Phanga T Maseko B Vansia D Kamtsendero L Gichane MW Contraceptive conversations among adolescent girls and young women and their partners, peers, and older female family members in Lilongwe, Malawi: a qualitative analysis. Stud Fam Plann. (2021) 52(4):397–413. 10.1111/sifp.12174 34585384 PMC8664985

[42] Ajayi AI Kabiru CW Otukpa E Ushie B Munthali A Thakwalakwa C Understanding the Experiences of Pregnant and Parenting Adolescents in Blantyre, Southern Malawi (2022).

[43] Chimbiri AM. The condom is an ‘intruder’in marriage: evidence from rural Malawi. Soc Sci Med. (2007) 64(5):1102–15. 10.1016/j.socscimed.2006.10.012 17240504

[44] Grundy SJ Maman S Graybill L Phanga T Vansia D Nthani T Intimate partner violence and contraception among adolescent girls and young women: a longitudinal analysis of the Girl Power-Malawi cohort. J Pediatr Adolesc Gynecol. (2022) 35(6):662–8. 10.1016/j.jpag.2022.06.005 35809851 PMC10071546

[45] Wigle JM Paul S Birn AE Gladstone B Kalolo M Banda L Participation of young women in sexual and reproductive health decision-making in Malawi: local realities versus global rhetoric. PLOS Glob Public Heal. (2022) 2(11):e0001297. 10.1371/journal.pgph.0001297 PMC10022123 36962663

[46] Kapira S. Assessing Barriers of Contraceptive Uptake among Adolescent Girls in a Rural District of Malawi. Boston: Harvard Medical School (2021).

[47] Makwinja AK Maida ZM Nyondo-Mipando AL. Delivery strategies for optimizing uptake of contraceptives among adolescents aged 15–19 years in Nsanje district, Malawi. Reprod Health. (2021) 18(1):15. 10.1186/s12978-020-01065-9 33472646 PMC7818728

[48] Yaya S Uthman OA Ekholuenetale M Bishwajit G. Women empowerment as an enabling factor of contraceptive use in sub-Saharan Africa: a multilevel analysis of cross-sectional surveys of 32 countries. Reprod Health. (2018) 15(1):214. 10.1186/s12978-018-0658-5 30572927 PMC6302468

[49] Bhushan NL Fisher EB Gottfredson NC Maman S Speizer IS Phanga T The mediating role of partner communication on contraceptive use among adolescent girls and young women participating in a small-group intervention in Malawi: a longitudinal analysis. Glob Public Health. (2022) 17(7):1392–405. 10.1080/17441692.2021.1924823 33977862 PMC8586050

[50] Jennings L Na M Cherewick M Hindin M Mullany B Ahmed S. Women’s empowerment and male involvement in antenatal care: analyses of demographic and health surveys (DHS) in selected African countries. BMC Pregnancy Childbirth. (2014) 14(1):1–11. 10.1186/1471-2393-14-297 25174359 PMC4161883

[51] Raru TB Ayana GM Zakaria HF Merga BT. Association of higher educational attainment on antenatal care utilization among pregnant women in east Africa using demographic and health surveys (DHS) from 2010 to 2018: a multilevel analysis. Int J Womens Health. (2022) 14:67–77. 10.2147/IJWH.S350510 35140524 PMC8819274

[52] Cardona C Rusatira JC Salmeron C Martinez-Baack M Rimon JG Anglewicz P Progress in reducing socioeconomic inequalities in the use of modern contraceptives in 48 focus countries as part of the FP2030 initiative between 1990 and 2020: a population-based analysis. Lancet Glob Heal. (2025) 13(1):e38–49. 10.1016/S2214-109X(24)00424-8 PMC11659844 39706659

[53] Hellwig F Barros AJD. Learning from success cases: ecological analysis of potential pathways to universal access to family planning care in low-and middle-income countries. Gates Open Res. (2023) 6:59. 10.12688/gatesopenres.13570.3 36726686 PMC9873636

[54] Hellwig F Ewerling F Coll CVN Barros AJD. The role of female permanent contraception in meeting the demand for family planning in low-and middle-income countries. Contraception. (2022) 114:41–8. 10.1016/j.contraception.2022.05.002 35568087

[55] G/Meskel AT Desta HO Bala ET. Factors associated with unmet need for family planning among married reproductive age women in Toke Kutaye district, Oromia, Ethiopia. Int J Reprod Med. (2021) 2021(1):5514498. 10.1155/2021/5514498 33855065 PMC8019393

[56] Bhushan NL. Social Influence and Contraceptive Use Among Adolescent Girls and Young Women in Malawi. Chapel Hill: The University of North Carolina at Chapel Hill (2018).

[57] Bornstein M Huber-Krum S Kaloga M Norris A. Messages around contraceptive use and implications in rural Malawi. Cult Health Sex. (2021) 23(8):1126–41. 10.1080/13691058.2020.1764625 32619393 PMC12928156

[58] Aviisah PA Dery S Atsu BK Yawson A Alotaibi RM Rezk HR Modern contraceptive use among women of reproductive age in Ghana: analysis of the 2003–2014 Ghana demographic and health surveys. BMC Womens Health. (2018) 18(1):141. 10.1186/s12905-018-0634-9 30126389 PMC6102847

[59] Komasawa M Yuasa M Shirayama Y Sato M Komasawa Y Alouri M. Demand for family planning satisfied with modern methods and its associated factors among married women of reproductive age in rural Jordan: a cross-sectional study. PLoS One. (2020) 15(3):e0230421. 10.1371/journal.pone.0230421 32187224 PMC7080244

