# Supplementary material for: Demand for and use of modern contraception among young women aged 15–24 years in Malawi: evidence from the Malawi demographic health survey, 2015–2016
Source: Front Reprod Health. 2025 Nov 24;7:1719985. doi: 10.3389/frph.2025.1719985 (PMC12682782; doi:10.3389/frph.2025.1719985)
Supplement: Supplementary file 1 [file Datasheet1.pdf]

Table 1: Operational definitions and coding of explanatory variables, Malawi DHS 2019–20

Variable	DHS code	Operational definition	Coding / Categories
Age group	v012	Current age of respondent	0 = 15–19 years; 1 = 20–24 years (ref.)
Residential area	v025	Type of place of residence	0 = Rural (ref.); 1 = Urban
Education level	v106	Highest level of schooling attended	0 = No education (ref.); 1 = Primary; 2 = Secondary; 3 = Tertiary
Wealth index	v190	Household wealth quintile (reclassified)	1 = Poor (poorest + poorer) (ref.); 2 = Middle; 3 = Rich (richer + richest)
Region	v024	Region of residence	1 = Northern; 2 = Central; 3 = Southern (ref.)
Age at first sex	v525	Age at first sexual intercourse (reclassified)	1 = <16 years (ref.); 2 = 16–19 years; 3 = 20+ years
Visited by health field worker	v393	Visited by fieldworker who discussed FP in last 12 months	0 = No (ref.); 1 = Yes
Attitude on domestic violence	v744a–v744e	Accepts wife-beating for any of 5 reasons (mean score = 0 if rejects all)	0 = Accepts some DV (ref.); 1 = Rejects all DV
Employment status	v714	Worked in last 12 months	0 = Not employed (ref.); 1 = Employed
Sexually active	v536	Had sexual intercourse in last 4 weeks	0 = No (ref.); 1 = Yes
Access to information	v157, v158, v159, v384a–c	Reads newspaper/magazine weekly OR listens to radio weekly OR watches TV weekly OR heard FP message on radio/TV/community meeting	0 = No (ref.); 1 = Yes
Parity	v201	Number of children ever born (categorised)	0 = 0 children (ref.); 1 = 1 child; 2 = 2–4 children; 3 = 5+ children
Marital status	v502	Current marital status	0 = Single (never married) (ref.); 1 = Married/cohabiting
Household head	v151	Sex of household head	0 = Male-headed (ref.); 1 = Female-headed
mDFPS (outcome)	v312, v624	Modern contraceptive demand satisfied (among women with demand)	0 = Demand not met by modern; 1 = Demand met by modern

Modern method use	v312	Current use of modern contraceptive method	0 = No; 1 = Yes (pill, IUD, injectables, implant, condom, female sterilization, LAM, emergency contraception, SDM)
Demand for FP	v312, v624	Has demand if using any method OR has unmet need	0 = No demand; 1 = Has demand
